# Activated hepatic stellate cells promote angiogenesis via interleukin-8 in hepatocellular carcinoma

**DOI:** 10.1186/s12967-015-0730-7

**Published:** 2015-11-22

**Authors:** Bing Zhu, Nan Lin, Min Zhang, Yong Zhu, Huanhuan Cheng, Shuxian Chen, Yunbiao Ling, Weidong Pan, Ruiyun Xu

**Affiliations:** Department of Hepatobiliary Surgery, The 3rd Affiliated Hospital of Sun Yat-sen University, No. 600 Tianhe Road, Guangzhou, 510630 Guangdong China; Department of Infectious Diseases, The 3rd Affiliated Hospital of Sun Yat-sen University, Guangzhou, China; Department of Gastrointestinal Surgery, The 4th Affiliated Hospital of Anhui Medical University, Hefei, China; Department of Ophthalmology, The 3rd Affiliated Hospital of Sun Yat-sen University, Guangzhou, China; Guangdong Provincial Key Laboratory of Liver Disease Research, Guangzhou, China

**Keywords:** Hepatocellular carcinoma, Activated hepatic stellate cells, Interleukin-8, Angiogenesis, Phospho-signal transducer and activator of transcription 3

## Abstract

**Background:**

Chemokines have been recognized as important modulators of angiogenesis, and they play critical roles in the development and metastasis of hepatocellular carcinoma (HCC), although their origins and latent molecular mechanisms remain elusive. The aim of this study was to investigate how activated hepatic stellate cells (a-HSCs) promote angiogenesis in HCC.

**Methods:**

A total of 22 HCC patients were enrolled randomly. We used immunohistochemistry, western blotting, and enzyme-linked immunosorbent assay (ELISA) to analyse the production of interleukin-8 (IL-8) in a-HSCs derived from HCC tissues. The angiogenic effects of IL-8 in vitro and in vivo were assessed by ELISA, real-time quantitative polymerase chain reaction, capillary tube formation assay, and chick embryo chorioallantoic membrane assay.

**Results:**

The present study showed that IL-8 was enriched predominantly in the tumour stroma of HCC tissues and was mainly derived from a-HSCs, rather than from hepatoma cells, in vivo and in vitro. Angiogenesis was most active at the invading edge, which was close to the a-HSCs. The angiogenic effect was dramatically attenuated by an IL-8 neutralizing antibody both in vitro and in vivo. Moreover, the IL-8 neutralizing antibody down-regulated Ser727-phosphorylated STAT3 levels in hepatoma cells treated with a-HSCs conditioned medium.

**Conclusions:**

These findings reveal that a-HSCs within the stroma of HCC contribute to tumour angiogenesis via IL-8.

**Electronic supplementary material:**

The online version of this article (doi:10.1186/s12967-015-0730-7) contains supplementary material, which is available to authorized users.

## Background

Hepatocellular carcinoma (HCC) is one of the most common solid malignant neoplasms and a leading cause of cancer-related death worldwide [1, 2]. Tumour progression relies on interactions between tumour cells and their surrounding microenvironment [3, 4]. The tumour microenvironment consists of stromal components, growth factors, proteolytic enzymes, extracellular matrix (ECM) proteins, and inflammatory cytokines [5, 6]. These elements play critical roles in tumour cell proliferation and survival as well as in angiogenesis and metastasis.

Angiogenesis plays a pivotal role in tumour growth and metastasis [7–9]. Dense tumour vasculature is associated with poor prognosis in certain types of cancer [10, 11]. Angiogenesis is regulated by transcription factors such as hypoxia inducible factor-1 (HIF-1), nuclear factor κB (NF-κB), and signal transducer and activator of transcription 3 (STAT3) [12]. In HCC, a net excess of angiogenic factors, including vascular endothelial growth factor (VEGF)-A, angiopoietins (Angs), and platelet-derived growth factor (PDGF)-B, is produced by tumour cells, vascular endothelial cells, immune cells, and pericytes [8, 13, 14].

The tumour microenvironment is composed of various cell types that influence the angiogenic response. Hepatic stellate cells (HSCs), the main type of stromal cell in the liver, are activated by tumour cells, inflammatory cytokines, and tumour-derived factors that are present in the surrounding milieu [15, 16]. Studies have suggested that activated HSCs (a-HSCs) play a crucial role in tumour angiogenesis and tumourigenesis [14, 17]. A-HSCs can promote the invasion of HCC cells by secreting matrix metalloproteinases (MMPs), urokinase-type plasminogen activator (uPA), interleukin-8 (IL-8), cyclooxygenase-2 (COX-2), and other factors [18–21].

IL-8, alternatively known as CXC motif ligand 8 (CXCL-8), is a proinflammatory CXC chemokine [22–24]. Its biological effects are mediated through binding to two cell surface G protein-coupled receptors, CXC motif receptor 1 (CXCR1) and CXCR2 [12, 25, 26]. Accumulating evidence has demonstrated that IL-8 is associated with tumour angiogenesis, metastasis, and poor prognosis in many types of cancer [27–30]. However, the precise mechanism by which IL-8 promotes tumour angiogenesis in HCC is not fully understood.

In this paper, we demonstrated that a-HSCs played an important role in angiogenesis associated with HCC. A-HSCs secreted IL-8, which in turn stimulated hepatoma cells (Hep3B and Huh-7) to express angiogenic factors, such as VEGF-A, to promote angiogenesis. Furthermore, we showed that a-HSCs promoted angiogenesis via IL-8 by up-regulating Ser727-phosphorylated STAT3 levels in hepatoma cells. These findings characterized certain interactions within the tumour microenvironment, and the data may help researchers develop more effective therapeutic strategies against HCC.

## Methods

### Patients and specimens

Pathologically confirmed HCC samples were obtained from 22 patients undergoing hepatectomy between 2013 and 2014 at the Department of Hepatobiliary Surgery of the Third Affiliated Hospital of Sun Yat-sen University in Guangzhou. These patients provided signed informed consent, and they had received no previous local or systemic treatment before the operation. Individuals with autoimmune disease, HIV or syphilis were excluded. The clinical stage was classified according to the guidelines of the International Union against Cancer. The clinical characteristics of all the patients are summarized in Additional file 1: Table S1. All the samples were anonymously coded in accordance with the local ethical guidelines (as stipulated by the Declaration of Helsinki). The experiments were conducted in strict accordance with a study design approved by the Clinical Research Ethics Committee at the Third Affiliated Hospital of Sun Yat-sen University in Guangzhou.

### Cell lines

Human hepatoma cell lines (Hep3B and Huh-7) were purchased from the Chinese Academy of Sciences. Primary HSCs were separated from the fresh tissue as described previously [31]. To minimize culture stress and clonal selection, HSCs passaged for up to 4-10 doublings were used for the experiments. Human umbilical vein endothelial cells (HUVECs) were isolated from fresh human umbilical cords with collagenase I (1 mg/mL; Sigma, USA) [32]. Only primary HUVECs at passages 3-6 were used in this study to avoid age-dependent cellular modifications [33].

### Capillary tube formation assay

Diluted Matrigel (Matrigel: Medium 199 = 1:2) (BD, Bioscience, USA) was added to a 96-well plate (50 mL/well) and allowed to polymerize for 2 h at 37 °C. HUVECs were then added at a density of 3 × 10^4^ cells per well in the 96-well plate with serum-free conditioned media from hepatoma cells, which were treated or not with HSC serum-free culture supernatant with or without the IL-8 neutralizing antibody (R&D Systems, USA) for 8 h in a 5 % CO_2_ incubator at 37 °C. Bloodvessel branch points were visualized by phase microscopy and quantitated in 3 random fields per well (10×) [34].

### Immunohistochemical staining

Paraffin-embedded and formalin-fixed samples were cut into 4-µm-thick sections, which were then processed for immunohistochemical staining as previously described [35].

### Immunofluorescence assay

A-HSCs growing in the 24-well flat-bottom plates were fixed, and stained with rabbit-anti-human monoclonal antibodies against alpha smooth muscle actin (α-SMA), vimentin and immunoglobulin (IgG, control) (Abcam, Cambridge, MA, USA) followed by treatment of AF488-conjugated donkey-anti-rabbit IgG (Molecular Probes, Carlsbad, CA, USA). The cell nuclei were counterstained with 4′-6-diamidino-2-phenylindole (Sigma-Aldrich, St. Louis, MO, USA) as previously described [36, 37]. These images were assessed using a fluorescence microscope (Leica DMI 4000B, Germany) at a wavelength of 488 nm and analysed with Leica Application suite software (version 4.0).

### Enzyme-linked immunosorbent assay (ELISA)

Concentrations of IL-8 and VEGF-A were detected by ELISA (R&D Systems, USA) as previously described [38]

### Chick embryo chorioallantoic membrane (CAM) assay

White leghorn chicken eggs on the fifth day after fertilization were purchased from the Animal Husbandry Institute of South China Agricultural University. The air chambers of these eggs were windowed after disinfection with 75 % alcohol. The shell membranes were then removed carefully, and a 50 μL droplet was pipetted onto the CAM. These windows were sealed with sterile ventilate surgical tapes, and the eggs were incubated without rotation at 80 % relative humidity and at 37.8 °C in the incubator. After 48 h, the results were observed under a ZEISS Lumar.V12 stereomicroscope.

### Multiplex bead–based enzyme–linked immunosorbent assay

According to the manufacturer’s instructions and a previously described method [39], conditioned supernatants were analysed using the multiplex bead–based Enzyme–Linked Immunosorbent Assay system (Millipore, Billerica, MA, USA). The results were analysed using a Luminex plate reader and the Milliplex analyst software (Luminex 200 System).

### Western blotting

For western blotting, proteins were separated by 10 or 15 % SDS-acrylamide gels and then transferred to 0.20 μM nitrocellulose membranes. After immunoblotting with an antibody against glyceraldehyde-phosphate dehydrogenase (GAPDH; Santa Cruz Biotechnology, USA), the protein expression of IL-8 and other markers was detected with an ECL kit. The antibodies included the following: NF-κB p65 (C22B4; #4764) and STAT3 (124H6; #9139), as well as their phosphorylated forms (Ser536-phosphorylated NF-κB p65: 93H1, #3033; Ser727-phosphorylated STAT3: #9134) (Cell Signaling, Beverly, MA, USA); HIF-1α (Cell Signaling, Beverly, MA, USA); and GAPDH and β-actin (Santa Cruz Biotechnology, USA).

### Real-time quantitative polymerase chain reaction (qPCR) analysis

Total RNA was extracted from cultured hepatoma cells using the TriPure Isolation Reagent (Roche Applied Science, Germany). The qPCR analysis of mRNA was executed according to the manufacturer’s instructions. The qPCR primer sequences corresponding to angiogenic factors are described in Table 1. All the reactions were performed in triplicate.

**Table 1:** Primer sequences for angiogenic factors

Factor	Primer sequence
VEGF-A	
Forward	5′-ATTCCCCACTTGAATCGGGC-3′
Reverse	5′-TCACTCACTTTGCCCCTGTC-3′
VEGF-B	
Forward	5′-AGGCTATATCCCAGTGGGGG-3′
Reverse	5′-ACAAGGGATGGCAGAAGAGC-3′
PDGF-A	
Forward	5′-CACTAAGCATGTGCCCGAGA-3′
Reverse	5′-AGATCAGGAAGTTGGCGGAC-3′
PDGF-B	
Forward	5′-ACTGATGGGGTCGCTCTTTG-3′
Reverse	5′-CAGGGATCAGGCAGGCTATG-3′
PDGF-C	
Forward	5′-GAGTCGCTGCTTCCAAAGTG-3′
Reverse	5′-TCTTGTACTCCGTTCTGTTCCTT-3′
Ang-1	
Forward	5′-AGCAACTGGAGCTGATGGAC-3′
Reverse	5′-TCCTCCCTTTAGTAAAACACCTTCT-3′

*VEGF* vascular endothelial growth factor, *PDGF* platelet-derived growth factor, *Ang-1* angiopoietin-1

### Statistical analysis

The data are presented as the mean ± standard error of the mean (SEM). The differences between groups were analysed using Student’s *t* test if only two groups were compared or using one-way analysis of variance (ANOVA) if more than two groups were compared. All the statistical tests were two-sided. All the experiments were performed at least three independent times. *P* values < 0.05 were considered statistically significant.

## Results

### A-HSCs secreted high levels of the inflammatory chemokine IL-8

**Fig. 1:**
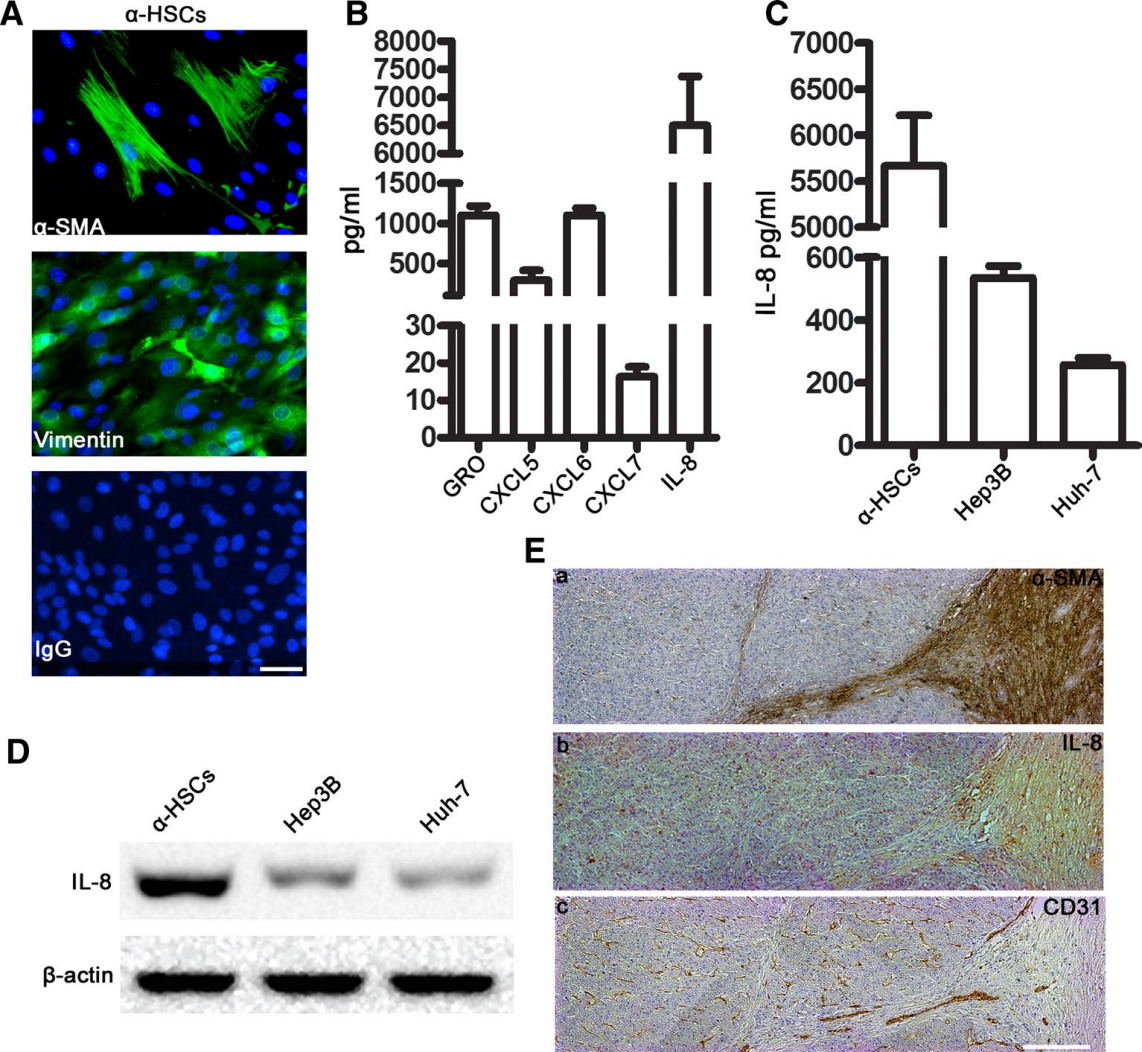
A-HSCs expressed high levels of IL-8. **A** Immunofluorescent staining of primary human a-HSCs isolated from a representative sample of HCC with an anti-α-SMA antibody,anti-vimentin antibody, and IgG. Scale bar, 50 μM. **B** The levels of various angiogenic chemokines in the cell-free culture supernatants of the a-HSCs were measured by Multiplex bead–based enzyme–linked immunosorbent assay at day 2. The data are expressed as mean ± SEM of triplicates. **(C)** The concentrations of IL-8 (pg/mL) in the supernatants of a-HSCs and hepatoma cells were determined by ELISA. The concentration of IL-8 in the a-HSCs culture medium was markedly higher compared with hepatoma cells. **(D)** The expression of IL-8 in the abovementioned cells was assessed by Western blotting. A-HSCs spontaneously released large amounts of IL-8, whereas hepatoma cells produced low levels of IL-8. **(E)** Hepatocarcinoma samples were stained with various antibodies, and different levels of antibodies expression can be seen on the same section. **E** (*a*) Immunohistochemical staining for α-SMA (1:1000) to identify a-HSCs in the tumour stromal region. **E** (*b*) The IL-8 distribution in hepatocarcinoma samples was visualized by immunohistochemical staining using the IL-8 neutralizing antibody (1:400). **E** (*c*) Neovascularization in hepatocarcinoma samples was visualized by immunohistochemical staining for CD31 (1:400). *Scale bar* 200 μM. One of the 22 representative micrographs is shown

To confirm that a-HSCs facilitate tumour angiogenesis, we first isolated a-HSCs from HCC tissues. A-HSCs were identified based on the high expression of the fibroblast-specific markers α-SMA and vimentin using fluorescence microscopy (Fig. 1A). Using the Multiplex bead-based Enzyme–Linked Immunosorbent Assay system, the levels of various inflammatory chemokines that are closely associated with angiogenesis, including GRO (α, β, and γ), CXCL-5, CXCL-6, CXCL-7 and IL-8, were detected in the 50 % a-HSC conditioned medium (HSC-CM) (CM:HSC-CM = 1:1) [23]. A-HSCs secreted significantly higher levels of IL-8 than of any other inflammatory chemokines (Fig. 1B).

It was reported that tumour cells also secret the angiogenic factor IL-8 [12]. Therefore, we compared the levels of IL-8 secreted by a-HSCs with those secreted by hepatoma cells. The ELISA assay revealed that the concentration of IL-8 in the a-HSC culture medium was markedly higher than that in the culture medium of hepatoma cells (Fig. 1C). Consistently, IL-8 production by a-HSCs and hepatoma cells was further confirmed by Western blotting (Fig. 1D).

### IL-8 was mainly enriched in the HCC stroma in vivo

To further study the role of IL-8 in tumour angiogenesis, we detected the distribution of IL-8 in tumour tissues from patients with HCC by immunohistochemistry. As shown in Fig. 1E (a and b), IL-8 was mainly enriched in the stroma surrounding the tumour, where numerous a-HSCs, as detected based on the fibroblast-specific marker α-SMA, were also present. This finding further confirmed that a-HSCs were the main source of IL-8 in HCC tissues. Furthermore, immunohistochemical staining for CD31 (Abcam, Cambridge, MA, USA), a microvessel marker, revealed that neovascularization occurred largely at the invading tumour edge, and close to the tumour stroma (Fig. 1E (c)).

### IL-8 neutralizing antibody suppresses tumour angiogenesis in vitro and in vivo

**Fig. 2:**
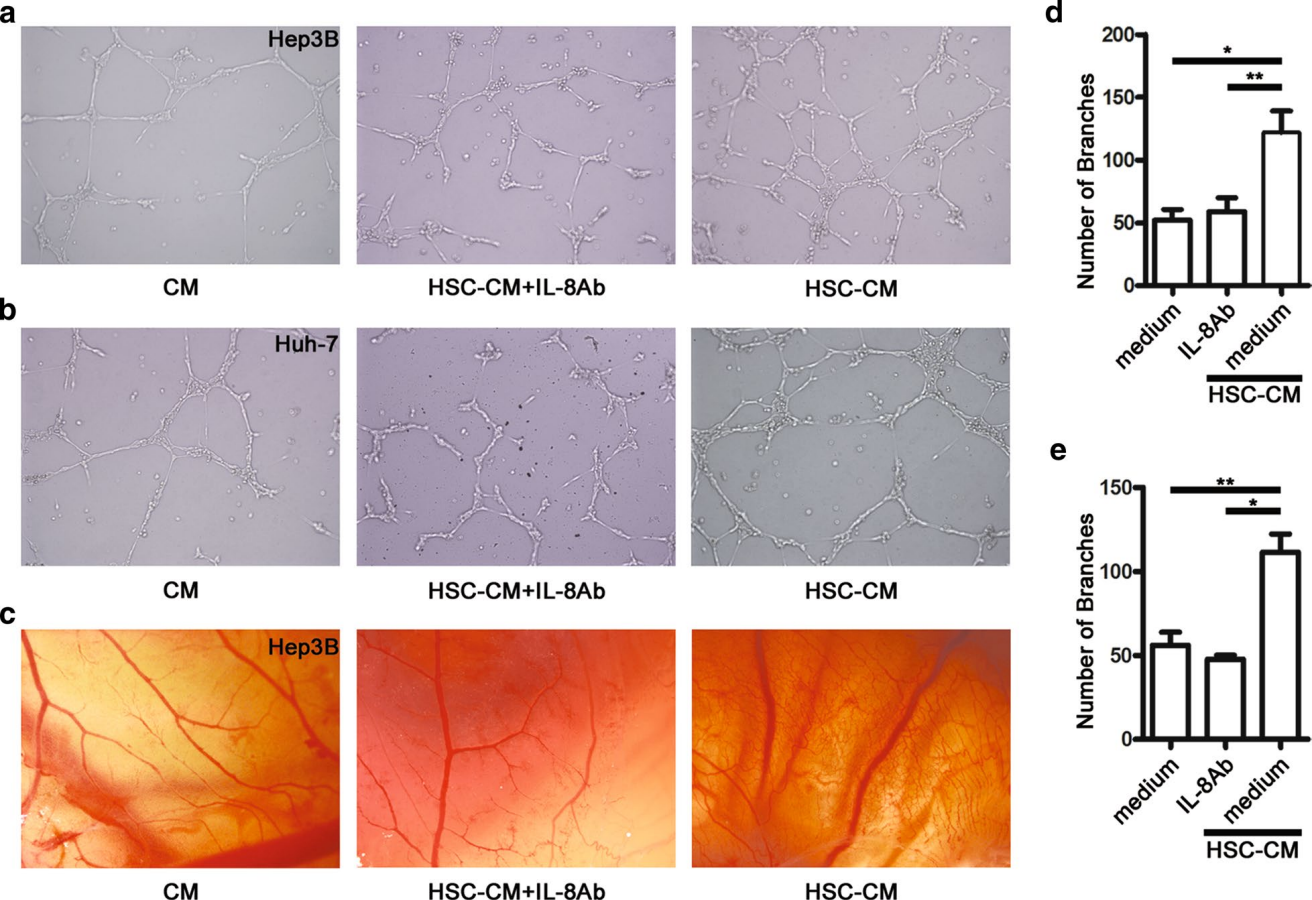
IL-8 neutralizing antibody repressed tumour angiogenesis in vitro and in vivo. **a**, **b** Soluble factors derived from HSC-CM-treated hepatoma cells induced angiogenic tube formation in vitro. The tube formation assay was done using HUVECs in the presence of serum-free conditioned medium from hepatoma cells, HSC-CM-treated hepatoma cells alone or supplemented with an IL-8 neutralizing antibody, and the IL-8 neutralizing antibody dramatically inhibited HUVEC tube formation. The illustrated results represent six separate experiments. **c** In the CAM assay, more capillary-like structures developed in the presence of serum-free supernatants from Hep3B cells exposed to the 50 % serum-free HSC-CM compared with supernatants from untreated Hep3B cells. The number of these capillary-like structures was significantly reduced by the IL-8 neutralizing antibody. **d**, **e** Representative images of the number of branch points generated by HUVECs in vitro. The data are presented as mean ± SEM of four independent experiments. *p < 0.05; **p < 0.01

To study the effect of IL-8 secreted by a-HSCs on angiogenesis, we collected the supernatants of a-HSCs and hepatoma cells cultured in the 50 % serum-free medium. Supernatants from untreated hepatoma cells afterculture for 24 h had only a slight effect on HUVEC tube formation. Supernatants from hepatoma cells that had been exposed to HSC-CM for 24 h significantly promoted angiogenic tube formation (Fig. 2a, b). Furthermore, the number of branch points within the capillary-like structures was dramatically reduced by the IL-8 neutralizing antibody (Fig. 2d, e).

A similar effect of a-HSCs and IL-8 on tumour angiogenesis was also observed in the CAM animal model (Fig. 2c). The group of eggs that was treated with HSC-CM showed more marked signs of angiogenesis compared to the other two groups in the CAM assay. Consistently, treatment with the IL-8 neutralizing antibody markedly inhibited angiogenesis.

### IL-8 neutralizing antibody down-regulated the expression of angiogenic factors in HSC-CM-treated hepatoma cells

**Fig. 3:**
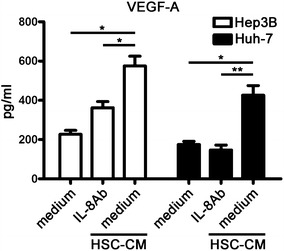
VEGF-A levels in the supernatant of hepatoma cells subjected to various treatments. The concentrations of VEGF-A (pg/mL) were assessed in the presence of serum-free conditioned medium from hepatoma cells, HSC-CM-treated hepatoma cells alone or supplemented with an IL-8 neutralizing antibody by ELISA. The IL-8 neutralizing antibody significantly down-regulated VEGF-A levels in the supernatant of hepatoma cells that were treated with 50 % HSC-CM for 24 h. The data are expressed as mean ± SEM of triplicates. *p < 0.05; **p < 0.01

**Fig. 4:**
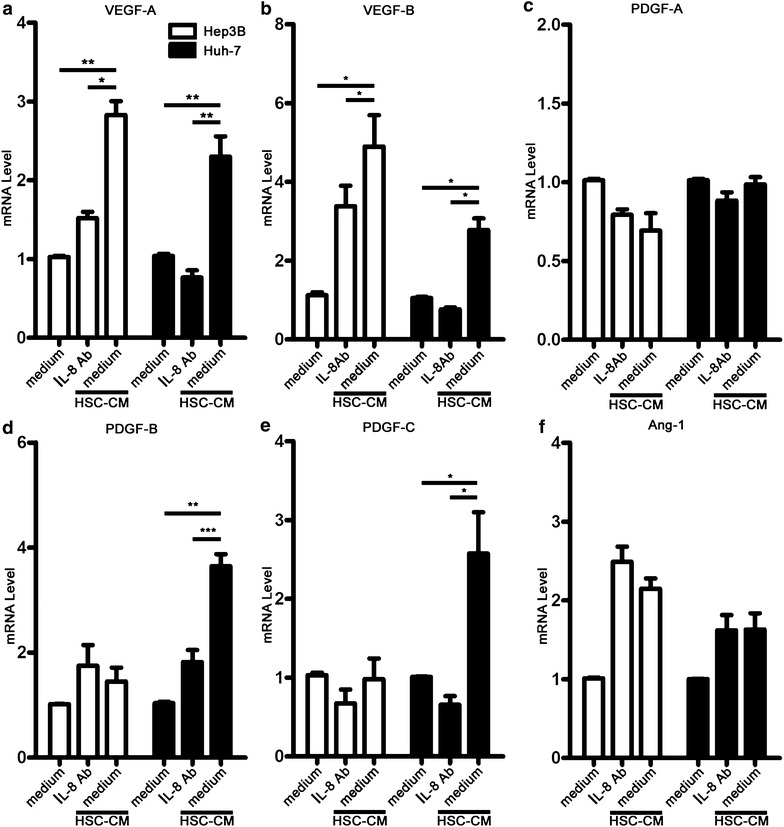
IL-8 neutralizing antibody down-regulated the mRNA expression of angiogenic factors in HSC-CM-treated hepatoma cells. **a**–**f** mRNA expression of angiogenic factors (VEGF-A, VEGF-B, PDGF-A, PDGF-B, PDGF-C, and Ang-1) in hepatoma cells after various treatments. In Hep3B cells, the mRNA levels of VEGF-A and VEGF-B were up-regulated in response to stimulation with HSC-CM and were down-regulated by IL-8 neutralizing antibody. The mRNA levels of PDGF-A, PDGF-B, PDGF-C, and Ang-1 remained unchanged in response to various conditioned media. In Huh-7 cells, the mRNA levels of VEGF-A, VEGF-B, PDGF-B, and PDGF-C were up-regulated in response to stimulation with HSC-CM and were down-regulated by IL-8 neutralizing antibody. The mRNA levels of PDGF-A and Ang-1 remained unchanged in response to various conditioned media. The data are presented as mean ± SEM of three independent experiments. *p < 0.05; **p < 0.01; ***p < 0.001

We next investigated the secretion of angiogenic factors in hepatoma cells under various conditions. VEGF-A levels were higher in the medium from HSC-CM-treated hepatoma cells (Hep3B and Huh-7) than from normal medium-treated hepatoma cells (Hep3B: *p* = 0.0193; Huh-7: *p* = 0.0155). Notably, the concentration of VEGF-A was dramatically decreased when the HSC-CM-treated hepatoma cells were treated with the IL-8 neutralizing antibody (Hep3B: *p* = 0.0152; Huh-7: *p* = 0.0065) (Fig. 3). Consistent with the above findings, HSC-CM up-regulated the levels of VEGF-A mRNA (*p* = 0.010) and VEGF-B mRNA (*p* = 0.034) in Hep3B cells compared to the normal medium (Fig. 4). Additionally, the IL-8 neutralizing antibody effectively down-regulated VEGF-A mRNA (*p* = 0.034) and VEGF-B mRNA (*p* = 0.048) levels. However, the mRNA levels of other angiogenic factors (PDGF-A, PDGF-B, PDGF-C and Angiopoietin-1 (Ang-1)) did not change significantly. In Huh-7 cells, the mRNA expression of angiogenic factors (VEGF-A (*p* = 0.008), VEGF-B (*p* = 0.031), PDGF-B (*p* = 0.007), and PDGF-C (*p* = 0.047)) was up-regulated in response to stimulation with HSC-CM. The mRNA levels of these angiogenic factors (VEGF-A (*p* = 0.005), VEGF-B (*p* = 0.0244), PDGF-B (*p* = 0.001), and PDGF-C (*p* = 0.022)) were significantly reduced by the IL-8 neutralizing antibody. Nevertheless, PDGF-A mRNA and Ang-1 mRNA levels remained unchanged in response to various conditioned media.

### IL-8 neutralizing antibody down-regulated Ser727-phosphorylated STAT3 levels in HSC-CM-treated hepatoma cells

**Fig. 5:**
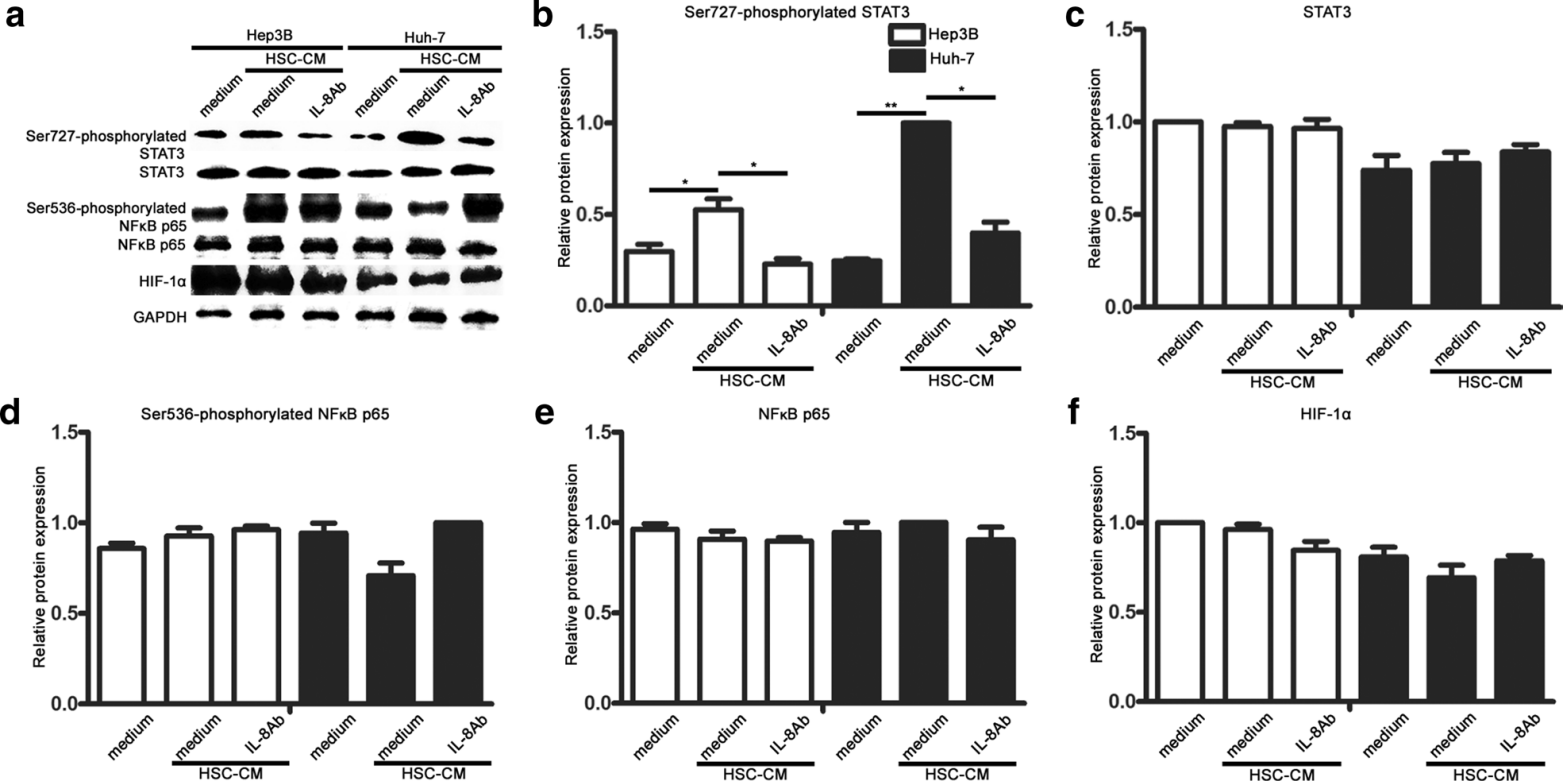
IL-8 neutralizing antibody exerted its function by suppressing Ser727-phosphorylated STAT3 signalling. **a** Hepatoma cells were cultured in CM alone (*lanes* *1* and *4*), 50 % HSC-CM (*lanes* *2* and *5*), or 50 % HSC-CM and the IL-8 neutralizing antibody (*lanes* *3* and *6*). Twenty-four hours after treatment, HIF-1α, Ser727-phosphorylated STAT3, and Ser536-phosphorylated NF-κB p65 levels were determined by Western blotting. **b**–**f** The results of Western blot analyses were quantified by densitometry (relative ratio to GAPDH). Western blotting showed differences in Ser727-phosphorylated STAT3 expression among the hepatoma cells after various treatments. Data are presented as mean ± SEM of three independent experiments. *p < 0.05; **p < 0.01. GAPDH was used as the internal control

Evidence suggests that IL-8 modulates tumour angiogenesis by up-regulating the expression of the HIF-1, NF-κB, and STAT3 transcription factors [12]. We used western blotting to analyse these signalling pathways in hepatoma cells exposed to HSC-CM (Fig. 5a). Amongst these pathways, only Ser727-phosphorylated STAT3 was activated in HSC-CM-treated hepatoma cells (Fig. 5b, c). Moreover, Ser727-phosphorylated STAT3 was effectively inhibited in HSC-CM-treated hepatoma cells by the IL-8 neutralizing antibody, whereas HIF-1α and NF-κB p65 activation was not affected (Fig. 5d–f). Taken together, these results demonstrated that a-HSCs partially exerted their angiogenic function via IL-8, which up-regulated Ser727-phosphorylated STAT3 levels in hepatoma cells.

## Discussion

Although a series of studies has indicated that a-HSCs play a key role in tumour angiogenesis, recurrence, and metastasis [31, 40, 41], the underlying mechanism remains incompletely understood. Our study demonstrated that a-HSCs produce a large amount of IL-8 in vitro; IL-8 was enriched predominantly in the tumour stroma rather than in the cancer nests of HCC samples. Furthermore, the IL-8 neutralizing antibody partially attenuated the ability of HSC-CM to induce angiogenesis and down-regulated Ser727-phosphorylated STAT3 levels in hepatoma cells. These findings revealed a distinct interaction between a-HSCs and hepatoma cells within the tumour microenvironment, which promoted tumour angiogenesis.

It is generally believed that the tumour stroma plays a key role in angiogenesis associated with HCC, and that a-HSCs are enriched predominantly in the tumour stroma [5, 18, 42]. However, the specific contributions of these a-HSCs to HCC angiogenesis have not been extensively studied. The present study provides evidence that a-HSC-derived IL-8 promoted angiogenesis in HCC. Several of our observations support this notion. First, a-HSCs were present primarily in the tumour stroma. Second, neovascularization was more active at the invading tumour edge, which is closer to the tumour stroma. Third, IL-8 was secreted in vitro mainly by a-HSCs, rather than by hepatoma cells. Finally, IL-8 was enriched predominantly in the tumour stroma, and the main distribution area of IL-8 corresponded with the location of a-HSCs in vivo.

IL-8 is a potent pro-inflammatory cytokine that plays a critical role in tumour angiogenesis, a crucial step in tumour progression [30, 43]. When CD1 nude mice bearing DLD-1^HIF−kd^ xenografts were treated with the IL-8 neutralizing antibody, the vessels were dramatically narrowed and fragmented, and the vascular density decreased [44]. In the current study, we observed that culture supernatants from HSC-CM-treated hepatoma cells significantly promoted angiogenic tube formation, and the angiogenic effects were significantly reduced by the IL-8 neutralizing antibody in vitro and in vivo. These results indicated that a-HSC-derived IL-8 is essential for HCC angiogenesis.

Previous studies have confirmed that IL-8 receptors (CXCR1 and CXCR2) are expressed on the surface of hepatoma cells [30]. IL-8 functions via its high-affinity receptors, CXCR1 and CXCR2 [26]. Evidence has indicated that IL-8 is closely associated with angiogenic factors during tumourigenesis [12, 45]. As a key mediator, IL-8 stimulates VEGF expression by activating the NF-κB pathway in endothelial cells [46]. Tumour angiogenesis is modulated by IL-8 via the up-regulated expression of the HIF-1, NF-κB, and STAT3 transcription factors [12]. However, in HIF-1-deficient tumours, IL-8 neutralization evokes a marked inhibition of tumour growth and angiogenesis [44]. In the current study, we found that IL-8 up-regulated angiogenic factors in hepatoma cells. Surprisingly, this process did not involve the transcription factor HIF-1α, NF-κB p65, Ser536-phosphorylated NF-κB p65, or STAT3, but instead required the activation of Ser727-phosphorylated STAT3. Our results provide direct evidence supporting the critical role of a-HSCs in HCC progression and reveal a fine-tuned collaboration between stromal cells and cancer cells in the tumour milieu.

The current understanding of the effect of a-HSCs on tumour angiogenesis strongly supports our findings [19, 40, 47]. Our research has some limitations. First, the 22 tumour samples evaluated in our study were not representative of all types of HCC, and the cohort was small considering the different conditions of these HCC patients. Second, further studies are needed to elucidate the molecular mechanism by which a-HSCs promote angiogenesis. Despite these shortcomings, our analysis shows that a-HSC-derived IL-8 plays a pivotal role in HCC-associated angiogenesis and reveals a fine-tuned collaboration between stromal cells and cancer cells in the tumour milieu. Larger studies are necessary to fully address this important question.

## Conclusions

Our results indicate that a-HSCs promote HCC angiogenesis via IL-8, which provides important new insights into the roles of a-HSCs in HCC progression. Understanding the mechanisms of a-HSC-mediated angiogenesis in HCC will aid in the rational design of promising targets for anti-tumour therapy.

## Additional file

10.1186/s12967-015-0730-7 Clinical Characteristics of the 22 HCC Patients.

## Abbreviations

HCC: hepatocellular carcinoma
a-HSCs: activated hepatic stellate cells
CXCL-8: C-X-C motif ligand 8
CXCR 1: C-X-C motif receptor 1
HUVECs: human umbilical vein endothelial cells
α-SMA: α-smooth muscle actin
IgG: immunoglobulin
ELISA: enzyme-linked immunosorbent assay
CM: conditioned medium
VEGF-A: vascular endothelial growth factor-A
CAM: chick embryo chorioallantoic membrane assay
GAPDH: glyceraldehyde-phosphate dehydrogenase
NF-κB: nuclear factor κB
STAT3: signal transducer and activator of transcription 3
HIF-1: hypoxia-inducible factor-1
qPCR: quantitative polymerase chain reaction
SEM: standard error of the mean
ANOVA: one-way analysis of variance
