# Supplementary material for: Activated hepatic stellate cells promote angiogenesis via interleukin-8 in hepatocellular carcinoma
Source: J Transl Med. 2015 Nov 22;13:365. doi: 10.1186/s12967-015-0730-7 (PMC4655083; doi:10.1186/s12967-015-0730-7)
Supplement: Supplementary file 1 — 10.1186/s12967-015-0730-7 Clinical Characteristics of the 22 HCC Patients. [file 12967_2015_730_MOESM1_ESM.docx]

**Supplementary Table 1.**

**Clinical Characteristics of the 22 HCC Patients**

| **Patient characteristics** | **Paraffin samples** |
| --- | --- |
| No. of HCC patients | 22 |
| Gender (male/female) | 17/5 |
| Age, years (median, range)  Tumor multiplicity (solitary/multiple) | 50, 31-78  19/3 |
| Intrahepatic metastasis (no/yes)  HbsAg (negative/positive) | 18/4  0/22 |
| Cirrhosis (absent/present) | 0/22 |
| Vascular invasion (absent/present) | 17/5 |
| TNM stage (I+II/III+IV) | 8/14 |
| Fibrous capsule (absent/present) | 14/8 |
| ALT, U/L (median, range) | 34, 18-123 |
| AFP, ng/ml (≤20/＞20) | 10/12 |
| IL-8 expression in stroma (negative/positive) | 9/13 |

**Abbreviations:** HbsAg, hepatitis B surface antigen; TNM, tumor node metastasis; ALT, alanine aminotransferase; AFP, α-fetoprotein; IL-8, interleukin-8.
